# Development of a Real-Time PCR Assay for the Detection of *Francisella* spp. and the Identification of *F. tularensis* subsp. *mediasiatica*

**DOI:** 10.3390/microorganisms12112345

**Published:** 2024-11-16

**Authors:** Alexandr Shevtsov, Ayan Dauletov, Uinkul Izbanova, Alma Kairzhanova, Nailya Tursunbay, Vladimir Kiyan, Gilles Vergnaud

**Affiliations:** 1 National Center for Biotechnology, Astana 010000, Kazakhstan; dauletov@biocenter.kz (A.D.); kairzhanova@biocenter.kz (A.K.); tursynbay@biocenter.kz (N.T.); vskiyan@gmail.com (V.K.); 2 Aikimbayev’s National Scientific Center for Especially Dangerous Infections, Almaty 050000, Kazakhstan; uincul71@mail.ru; 3 Institute for Integrative Biology of the Cell (I2BC), Centre National de la Recherche Scientifique (CNRS), Commissariat à l’Énergie Atomique (CEA), Université Paris-Saclay, 91198 Gif-sur-Yvette, France

**Keywords:** real-time PCR, *Francisella* spp., *Francisella tularensis* subsp. *mediasiatica*, ISFtu1, ISFtu2

## Abstract

Tularemia is an acute infectious disease classified as a natural focal infection, requiring continuous monitoring of both human and animal morbidity, as well as tracking of pathogen circulation in natural reservoirs and vectors. These efforts are essential for a comprehensive prevention and containment strategy. The causative agent, *Francisella tularensis*, comprises three subspecies—*tularensis*, *holarctica*, and *mediasiatica*—which differ in their geographic distribution and virulence. The ability to directly detect the pathogen and differentiate between subspecies has enhanced diagnostics and allowed a more accurate identification of circulation areas. Real-time PCR protocols for identification of *F. tularensis* subspecies *tularensis* and *holarctica* have been developed, utilizing specific primers and probes that target unique genomic regions. In this study, we present the development of a new real-time PCR assay for the detection of *Francisella* spp. and differentiation of *F. tularensis* subsp. *mediasiatica*. The specificity of the assay was tested on DNA from 86 bacterial species across 31 families unrelated to *Francisella* spp., as well as on DNA collections of *F. tularensis* subsp. *mediasiatica* and *F. tularensis* subsp. *holarctica*. The limit of detection (LOD95%) for real-time PCR in detecting *Francisella* spp. was 0.297 fg (0.145 genomic equivalents, GE) for *holarctica* DNA and 0.733 fg (0.358 GE) for *mediasiatica* DNA. The LOD95% for subspecies differential identification of *mediasiatica* was 8.156 fg (3.979, GE). The high sensitivity and specificity of these developed protocols enable direct detection of pathogens in biological and environmental samples, thereby improving the efficiency of tularemia surveillance in Kazakhstan.

## 1. Introduction

Natural focal diseases represent a large group of illnesses characterized by the continuous circulation of pathogens in natural landscapes, sustained by the presence of natural hosts or reservoirs. For transmissible diseases, this also includes carriers [1]. Globally, there are more than 180 types of natural focal diseases, encompassing viral, bacterial, fungal, protozoal, and helminthic origins [2]. These diseases are significant public health concerns [3]. Continuous monitoring of human and animal morbidity, as well as tracking of pathogen circulation in natural reservoirs and carriers, are critical components of a comprehensive strategy to prevent and contain natural focal infections [4].

Tularemia is a natural focal acute febrile zoonotic disease caused by the highly contagious Gram-negative coccobacillus *Francisella tularensis* [5]. Tularemia is characterized by a variety of clinical manifestations, which are associated with the routes of infection. There are six classical clinical forms, including ulcero-glandular, glandular, ocular, oropharyngeal, pneumonic, and typhoidal forms [6,7]. The severity of tularemia largely depends on the subspecies and subpopulation of the pathogen [8]. The number of host species susceptible to infection by this agent (>100 species) is higher than for any other known zoonotic pathogen [9]. Recently, more data have emerged on the significant role of water sources in maintaining the infection and in infecting people [10]. In 1932, Japanese scientists initiated research on the possibility of using *F. tularensis* as a biological weapon [11]. For many years, *F. tularensis* has been considered a potential biological weapon and was part of the offensive programs of both the United States and the Soviet Union [12]. The diversity of naturally susceptible animals, climate change and the resulting expansion of tick distribution areas, the involvement of water sources, and the emergence of new tularemia foci necessitate coordinated efforts among medical, veterinary, and biological specialists to control tularemia [13].

Tularemia is listed among the natural focal infections that are continuously monitored in Kazakhstan to assess the incidence in wild animals and the circulation of pathogens in vectors and reservoirs [14]. The total area of tularemia foci in Kazakhstan covers approximately one-fifth of the country (552,000 km^2^) [15] and is endemic to two of the three subspecies, *F. tularensis* subsp. *holarctica* and *F. tularensis* subsp. *mediasiatica*. Subspecies *mediasiatica* is the least studied due to its limited distribution and the absence of reported human infections. The recent detection of new foci of *F. tularensis* subsp. *mediasiatica* in the Altai and Krasnoyarsk regions of Russia, along with findings suggesting its intermediate virulence between *F. tularensis* subsp. *tularensis* and *holarctica* in laboratory animals, has renewed scientific interest in a comprehensive study of this pathogen [16,17]. Understanding the origin of subspecies *mediasiatica* might be key to understanding the emergence of the two other subspecies.

Areas endemic to tularemia undergo annual epidemiological monitoring, which includes the collection and laboratory testing of samples from rodents and ticks. Over the past twenty years, approximately 20,000 rodents and 220,000 ticks have been tested for tularemia in Kazakhstan using microbiological and serological methods [18].

Monitoring studies that employ microbiological methods are essential for isolating pure bacterial cultures and differentiating between subspecies and subpopulations, which vary in pathogenicity, virulence, and infection severity. By identifying and understanding these differences, researchers can more accurately assess outbreak risks and forecast their potential impact, particularly when an epizootic is detected among carriers or when the activity of a focus intensifies. Subspecies and subpopulation differentiation within clinical samples enables prediction of the disease outcome and severity in patients, providing valuable information for effective treatment strategies. For laboratory personnel, understanding these distinctions is crucial to gauging the safety measures needed when handling clinical isolates, thereby enhancing laboratory safety and optimizing resource allocation [19].

Biochemical identification of subspecies is indeed labor-intensive, as it requires cultivating live cultures—a process that significantly heightens the risk of laboratory-acquired infections [20,21,22]. In light of these challenges, DNA-based methods for subspecies identification of *F. tularensis* have become highly valued in laboratory practice. They offer several advantages, including enhanced safety for laboratory personnel since they do not require handling live cultures, and low cost long-term storage owing to DNA stability, which is crucial for genotyping in outbreak monitoring and population analysis. Pulsed-field gel electrophoresis (PFGE) and multiple-locus variable-number tandem repeat (VNTR) analysis (MLVA) were initially used for *F. tularensis* subtyping. These methods allow reliable identification of subspecies, including distinguishing *F. tularensis* subsp. *tularensis* strains into A-west (A.II) and A-east (A.I) types. However, PFGE has not gained wide adoption due to several limiting factors. DNA preparation for PFGE is complex, the procedure is time-consuming, and data exchange between laboratories is difficult, necessitating specific equipment and specialized software. Consequently, simpler, safer, and more accessible DNA-based alternatives continue to be in demand [23]. MLVA indeed provides high discriminatory power for distinguishing among clonal pathogens like *F. tularensis*, making it valuable in epidemiological studies and outbreak tracking. However, a limitation of MLVA is the occurrence of homoplasy at VNTR loci, where independent mutations can lead to the same repeat patterns in unrelated strains [24]. Given the high sequence identity among *F. tularensis* subspecies and the genomic rearrangements caused by homologous recombination at insertion sequence elements ISFtu1 and ISFtu2, insertion–deletion (INDEL) polymorphism analysis has emerged as a valuable approach for subspecies differentiation. In 2007, Pär Larsson and colleagues introduced a subspecies identification method based on loci showing INDEL polymorphism. Through the examination of five genomes, they identified 280 INDEL markers, with 38 showing significant polymorphism suitable for differentiating subspecies. This INDEL typing approach effectively separated *F. tularensis* subspecies, delineated clades A.I and A.II within subspecies *tularensis*, and distinguished Japanese strains from other *holarctica* strains [24]. A recent development in subspecies differentiation of *F. tularensis* introduced a typing scheme utilizing five highly informative INDEL markers. This streamlined approach provides a targeted method for distinguishing among *F. tularensis* subspecies, as well as for differentiating between the A.I and A.II subpopulations within subspecies *tularensis* [25]. A subspecies differentiation scheme has been developed that utilizes a single primer designed to target “hot spots” of intragenomic recombination. This method generates a pattern of six distinct PCR fragments, with the specific combination of these fragments enabling accurate differentiation between subspecies of *F. tularensis* and *F. novicida* [26,27]. The primary limitation of these existing differentiation methods lies in their reliance on PCR followed by electrophoretic or capillary separation, while many laboratories engaged in monitoring natural focal infections are primarily equipped with real-time PCR (qPCR) technology. A qPCR approach has been developed for differentiating subpopulations of *F. tularensis* A.I and A.II, subspecies *holarctica*, and *F. novicida*, employing three markers in a multiplex reaction. However, this protocol has not yet been validated for use with *F. tularensis* subspecies *mediasiatica* [28]. An identification scheme using eleven single nucleotide polymorphisms (SNPs) enables precise differentiation of *F. tularensis* from *F. novicida*, *F. hispaniensis*, *F. philomiragia*, and *Francisella*-like endosymbionts, as well as differentiation into subspecies and specific subpopulations of *F. tularensis*. This approach requires a hierarchical framework for proper interpretation, which can complicate analysis. Additionally, SNPs within primer or probe regions can lead to nonspecific results [29]. A further limitation of these methods is their moderate sensitivity, restricting their application primarily to identification of isolated strains and reference collection strains rather than being effective for direct detection in complex environmental or clinical samples.

The most effective and widely used methods are those with high sensitivity that rely on a single marker to differentiate subspecies or subpopulations. This approach offers the flexibility for researchers to employ a combination of protocols tailored to the endemic characteristics of the region. With its high sensitivity, qPCR has become a valuable tool for first-line diagnostics in monitoring studies. A qPCR protocol using two markers to differentiate the *F. tularensis* subspecies *holarctica* and *tularensis* has been proposed [30]. The sensitivity of this method allows the direct differentiation of pathogens in environmental samples, as well as in rodent and tick specimens, which are important vectors in the epidemiology of tularemia [31,32,33]. The proposed qPCR testing algorithm, which utilizes eight pairs of primers and TaqMan probes, represents a more comprehensive method for the identification and differentiation of *F. tularensis* subspecies and related species. This algorithm allows for the differentiation of *F. tularensis* subsp. *tularensis* subtypes AI and A.II, *F. tularensis* subsp. *holarctica*, *F. tularensis* subsp. *mediasiatica*, *F. novicida*, and closely related species such as *F. philomiragia*, *F. persica*, and *Francisella*-like endosymbionts. Incorporating a 16S rRNA amplification as positive control ensures that any potential inhibition of the PCR reaction can be assessed, adding an extra layer of reliability to the results. The primers and TaqMan probes can be multiplexed, allowing for simultaneous detection of multiple targets in a single reaction. The high sensitivity of this qPCR protocol makes it suitable for both epidemiological monitoring and clinical applications [34].

The development of PCR tests for detecting *Francisella* spp. and differentiating, subsp. *mediasiatica* across all sublineages is crucial due to the observed limitations of the previously proposed qPCR algorithm. The algorithm demonstrated high sensitivity and reliability for identifying various *Francisella* species and subspecies, including the M.I lineage of *F. tularensis* subsp. *mediasiatica*. However, as shown by the Primer-BLAST analysis, the proposed primers only generate target fragments in the M.I lineage genomes (genome sequence accessions CP130000, CP130001, and CP000915) and fail to generate fragments in the M.II and M.III lineage genomes (e.g., CP129999, CP129998, and more recent genomes deposited in 2023). These findings suggest that the current algorithm may not be applicable for identifying *F. tularensis* subsp. *mediasiatica* in regions endemic for lineages M.II and M.III, which highlights the need for additional PCR testing strategies. The primary goal of this work is to address these limitations by developing PCR tests capable of detecting and differentiating *F. tularensis* subsp. *mediasiatica* from all currently known sublineages, ensuring broader applicability across various endemic regions.

## 2. Materials and Methods

### 2.1. DNA Samples

The DNA samples used in this study were from *F. tularensis* subsp. *mediasiatica* and *F. tularensis* subsp. *holarctica*. Bacteria were cultured, and DNA was isolated in the BSL-3 laboratory at the Masgut Aikimbayev National Scientific Center for Especially Dangerous Infections (NSCEDI) in Almaty, Kazakhstan, under the Ministry of Healthcare of the Republic of Kazakhstan. The *F. tularensis* strains were cultured, inactivated, and DNA was isolated using the QIAamp DNA Mini Kit (Qiagen, Hilden, Germany), following the protocols described previously [35].

The study also utilized a bacterial DNA collection from the Applied Genetics Laboratory at the National Center for Biotechnology, which was established during the provision of species identification services based on 16S rRNA sequencing. Bacterial genomic DNA was isolated using either the QIAamp DNA Mini Kit (Qiagen, Hilden, Germany) or the Wizard^®^ Genomic DNA Purification Kit (Promega Corporation, Madison, WI, USA). Species identity was confirmed through 16S rRNA sequencing using the primers 8F (5′-AGAGTTTGATCCTGGCTCAG) and 806R (5′-GGACTACCAGGGTATCTAAT) [36]. Sequencing was performed with the BigDye Terminator v3.1 Cycle Sequencing Kit (Applied Biosystems, Vilnius, Lithuania) on a DNA Analyzer 3730xl (Applied Biosystems, Hitachi, Japan, Tokyo). Identification was carried out using NCBI BLAST with the nr/nt database. Genomic DNA from cattle, sheep, and mice was isolated from muscle tissue using the Wizard^®^ Genomic DNA Purification Kit (Promega Corporation, Madison, WI, USA).

### 2.2. Primer Development

To search for genetic differences between *F. tularensis* subspecies, multiple alignments of conserved genomic sequences with rearrangements of the genomes—NZ_CP058276 *F. tularensis* subsp. *tularensis* isolate FSC237, NZ_CP098826 *F. tularensis* subsp. *holarctica* strain A-1341, CP000915 *F. tularensis* subsp. *mediasiatica* strain FSC147, and NZ_CP021490 *F. novicida* strain TCH2015—were performed using MAUVE [37] as implemented within Geneious Prime (Java Version 11.0.20.1+1, GraphPad Software LLC, Boston, MA, USA). MAUVE alignment was performed using the progressive MAUVE algorithm with default parameters. We selected primers from a region identical across all subspecies of *F. tularensis* and *F. novicida*, yet differing in *F. tularensis* subsp. *mediasiatica*. Such regions are typically associated with the integration of mobile genetic elements. The distinctive region was randomly assigned 25 bp primers and used in Primer-BLAST. In cases where unique target fragments were predicted using only two primers in *mediasiatica* genomes, this region was utilized for subsequent primer and TaqMan probe design. An alignment of the target region from the three subspecies of *F. tularensis* and *F. novicida* was conducted. From the genomes of the subspecies *tularensis*, *holarctica*, and of *F. novicida*, the homologous region from *F. tularensis* subsp. *mediasiatica* and an additional 300 bp flanking this region were utilized. The sequences were aligned in BioEdit (version 7.2.5) [38]. TaqMan probe and primers were selected for the unique region of *F. tularensis* subsp. *mediasiatica*.

Primers for detecting *Francisella* spp. were designed based on the conserved nucleotide sequence of the insertion sequence (IS) element ISFtu2. An alignment of the sequences from this region across the three subspecies of *F. tularensis*, as well as *F. novicida*, *F. philomiragia*, *F. hispaniensis*, *Francisella* sp. TX077308, *Francisella* sp. LA112445 strain LA11-2445, and *F. uliginis* strain TX07-7310, was conducted. Primer characteristics were evaluated using Lasergene Primer Select 6.1 (DNASTAR, Madison, WI, USA). Primer specificity was assessed against the NCBI “nt” and “RefSeq representative genomes” databases using Primer-BLAST (https://www.ncbi.nlm.nih.gov/tools/primer-blast, accessed on 5 February 2024) [39].

### 2.3. Conducting PCR

PCR for the detection of *Francisella* spp. and subspecies differentiation of *F. tularensis* subsp. *mediasiatica* was performed in separate reaction mixtures. Each real-time PCR was conducted in a total volume of 25 µL, consisting of 12.5 µL BioMaster UDG-HS-qPCR-2x (Biolabmix, Novosibirsk, Russia), primers and probes at the concentrations specified in Table 1, DNA, and UltraPure™ DNase/RNase-Free Distilled Water (Invitrogen, Carlsbad, CA, USA). Distilled water was used as negative control. All thermal cycling steps were carried out in a QuantStudio™ 5 Real-Time PCR system (Applied Biosystems, Singapore). The PCR protocol included the following steps: an anticontamination treatment at 50 °C for 2 min, pre-denaturation at 95 °C for 5 min, followed by 10 cycles of denaturation at 95 °C for 15 s and annealing/extension at 60 °C for 1 min (without detection of fluorescent signal). This was followed by 35 cycles of denaturation at 95 °C for 15 s and annealing/extension at 60 °C for 1 min (where results were recorded).

### 2.4. Amplification Efficiency (AE)

The efficiency of PCR amplification was assessed using a series of diluted *F. tularensis* subsp. *mediasiatica* and *F. tularensis* subsp. *holarctica* DNA samples. Genomic equivalents were calculated using the Copy Number Calculator (https://www.technologynetworks.com/tn/tools/copynumbercalculator, accessed on 16 April 2024). DNA concentration was measured with a Qubit 2.0 fluorimeter (Life Technologies, Tecan, Grödig, Austria) using the Qubit dsDNA HS Assay Kit (Invitrogen™, Carlsbad, CA, USA). The following DNA dilution series were used: 2.05 ng (1,000,000 GE), 0.12 ng (62,500 GE), 0.008 ng (3906 GE), 0.0005 ng (244 GE), 31.2 fg (15.2 GE), and 1.95 fg (0.95 GE). Each dilution was tested three times. Real-time PCR was performed using a QuantStudio™ 5 Real-Time PCR Instrument (Applied Biosystems, Singapore), and the results were analyzed with QuantStudio™ Design and Analysis Software v1.5.3 (Thermo Fisher Scientific, Waltham, MA, USA).

### 2.5. Analytical Sensitivity

In the first stage, the maximum sensitivity of the developed tests was assessed using a series of *F. tularensis* subsp. *mediasiatica* and *F. tularensis* subsp. *holarctica* DNA samples. Each DNA sample concentration was measured three times with a Qubit 2.0 fluorimeter (Life Technologies, Tecan, Grödig, Austria) using the Qubit dsDNA HS Assay Kit (Invitrogen™, Carlsbad, CA, USA). The average concentration measure was used to prepare a series of fourfold DNA dilutions. The dilution range varied from 2.05 ng per reaction, approximately equivalent to 1,000,000 genomic equivalents (GE), down to 0.031 fg, or 0.0015 GE. Each dilution was prepared in triplicate.

Based on the sensitivity limit data, three-fold DNA dilutions were prepared to estimate the detection limit (LOD) with 95% probability. DNA samples of the *mediasiatica* and *holarctica* subspecies were diluted three-fold, starting from a concentration of 2.05 pg (approximately 1000 GE) down to 0.0116 fg (0.006 GE). Each dilution was performed in seven replicates for one experiment, which was repeated five times on different days, resulting in a total of 35 replicates for each concentration. DNA dilution was repeated before each experiment. Probit analysis in R software version 4.1.2 was used for the statistical analysis of the minimum detectable concentration (LOD95). The model was constructed using the dose.p function from the MASS package in R 3.6.2 [40].

### 2.6. Specificity Analysis

Specificity was confirmed using a collection of DNA samples from 86 bacterial species belonging to 31 families (Table S1) that are not significantly related to *Francisella* spp., as well as from a collection of previously genotyped strains of *F. tularensis* subsp. *mediasiatica* (28 strains) and *F. tularensis* subsp. *holarctica* (39 strains) [35,41]. Among higher organisms, genomic DNA was obtained from mice, cattle, and sheep. The DNA concentration of *F. tularensis* subsp. *mediasiatica* and *F. tularensis* subsp. *holarctica* strains was measured using the Qubit dsDNA HS Assay Kit (Invitrogen™, Carlsbad, CA, USA), normalized to a single concentration, and 1 ng was added to the PCR reaction. The DNA of the remaining bacterial strains, as well as animal DNA, was measured using the Qubit dsDNA BR Assay Kit (Invitrogen™, Carlsbad, CA, USA). The amount of DNA used in the reaction is specified in Table S1.

## 3. Results

### 3.1. Development of Primers for Detection of Francisella spp. and Subspecies Identification of Mediasiatica

Universal primers for *Francisella* spp. were designed targeting conserved sequences of the mobile element insertion sequence (IS) element ISFtu2 (Table 1). In silico specificity testing of the primers selected for ISFtu2 using BLAST (https://www.ncbi.nlm.nih.gov/tools/primer-blast, accessed on 5 February 2024) predicted that they annealed specifically to species within the genus *Francisella* and will produce a 153 bp PCR product.

**Figure 1:**
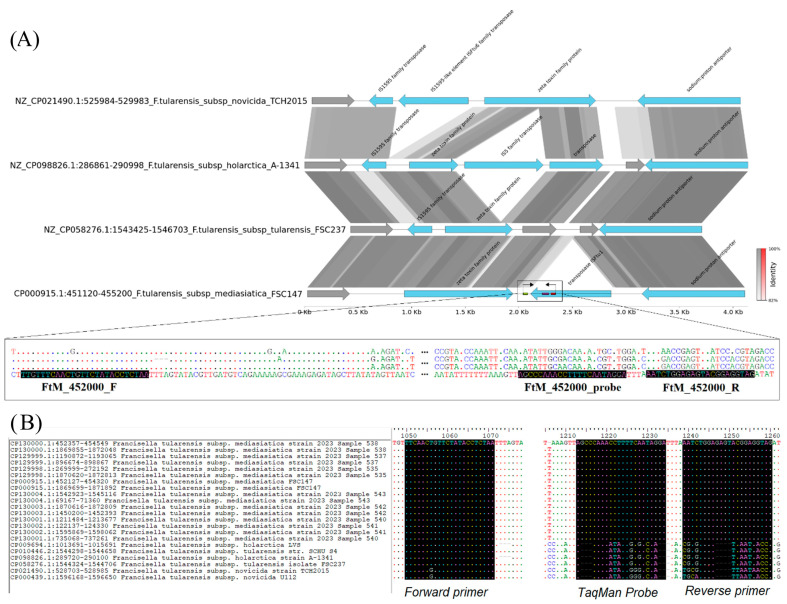
Primers design for identification of *F. tularensis* subsp. *mediasiatica.* (**A**) Aligned genomic fragments of *F. novicida* and of the three *F. tularensis* subspecies representatives used for primer selection for the subspecies identification of *mediasiatica*. (**B**) The alignment of target fragments from all circular genomes of the *F. tularensis* subsp. *mediasiatica* and two sequences from the genomes of the subspecies *holarctica*, *tularensis*, and *F. novicida*.

The analysis of genomic data from *F. novicida* TCH2015, *F. tularensis* subsp. *holarctica* A-1341, *F. tularensis* subsp. *tularensis* FSC237, and *F. tularensis* subsp. *mediasiatica* FSC147 enabled the identification of a sequence configuration at position 452,023 to 453,951 bp in FSC147 (nucleotide accession CP000915.1) unique to *mediasiatica* FSC147 strain. In this region, a mobile element (ISFtu1) integrated after the gene encoding the zeta toxin family protein (Figure 1A). Based on the aligned sequences, two PCR primers and a fluorescent probe were selected (Table 1).

Based on the Primer-BLAST results, the PCR primers designed to identify the subspecies *F. tularensis* subsp. *mediasiatica* are predicted to generate two target products of 215 bp (214 bp in the CP130000 genome) in *mediasiatica* genomes in addition to three products of about 2360 bp generated by the reverse primer alone. In the genomes of the subspecies *holarctica* and *tularensis*, the reverse primer alone is predicted to produce from one to three PCR fragments of at least 1700 bp in length. Of the eight complete genomes of *F. tularensis* subsp. *mediasiatica* (Table S2), two DNA regions with annealing sites of the selected primers were extracted. From the *holarctica* and *tularensis* genomes, as well as from the *F. novicida* genomes, a region complementary to the forward primer with an additional 300 bp from 3′ was extracted. As a result of the alignment, it was found that the primers and TaqMan probe were selected to a conserved sequence in all eight strains of *F. tularensis* subsp. *mediasiatica* (Figure 1B). These strains belong to sublines M.I–M.III.

### 3.2. Analytical Sensitivity and Amplification Efficiency

**Figure 2:**
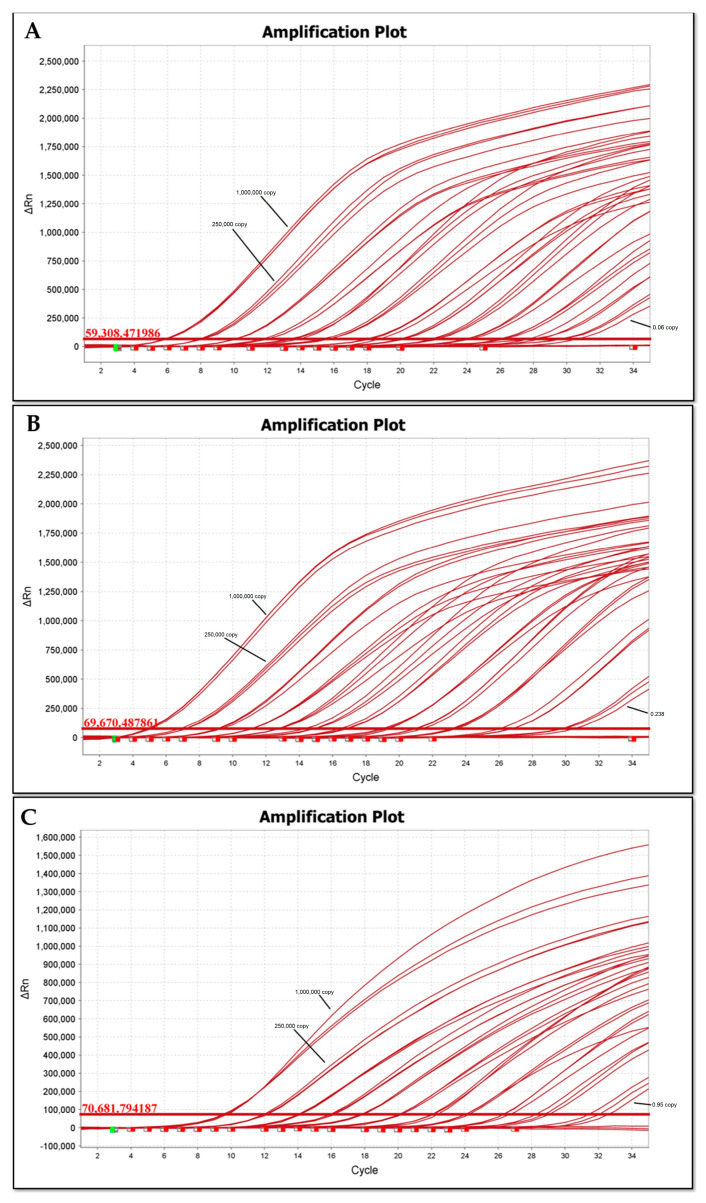
Sensitivity testing of real-time PCR for *Francisella* spp. detection and subspecies differentiation of *F. tularensis* subsp. *mediasiatica*. qPCR assays were performed on *Francisella* spp. samples using 4-fold serial dilutions ranging from 1,000,000 to 0.015 copies. (**A**) Real-time PCR results for the detection of *Francisella* spp. using *F. tularensis* subsp. *holarctica* DNA (Primers/TaqMan: isftu-2_F_242, isftu-2_R_396 and isftu-2_Probes_331). (**B**) Real-time PCR results for the detection of *Francisella* spp. using *F. tularensis* subsp. *mediasiatica* DNA (Primers/TaqMan: isftu-2_F_242, isftu-2_R_396 and isftu-2_Probes_331). (**C**) Real-time PCR results for the subspecies differentiation of *F. tularensis* subsp. *mediasiatica* (Primers/TaqMan: FtM_452000_F, FtM_452000_R and FtM_452000_probe). Each sample was tested in triplicate. The horizontal red line indicates the fluorescence threshold.

The maximum sensitivity of the real-time PCR detection for *Francisella* spp. was determined to be 0.122 fg (0.06 GE) for *holarctica* and 0.488 fg (0.238 GE) for *mediasiatica*. This is in agreement with the relative copy number of ISFtu2 elements in the two subspecies. For subspecies differentiation of *mediasiatica*, the sensitivity was 1.96 fg (0.95 copies) in agreement with the amplification of a single-copy target (Figure 2). Accordingly, Probit analysis results indicated that the LOD95% (limit of detection with 95% probability) for real-time PCR detection of *Francisella* spp. was 0.297 fg (0.145 GE) for *holarctica* DNA and 0.733 fg (0.358 GE) for *mediasiatica* DNA. For subspecies identification of *mediasiatica*, the LOD95% was 8.156 fg (3.979 GE) (Figure S1).

**Figure 3:**
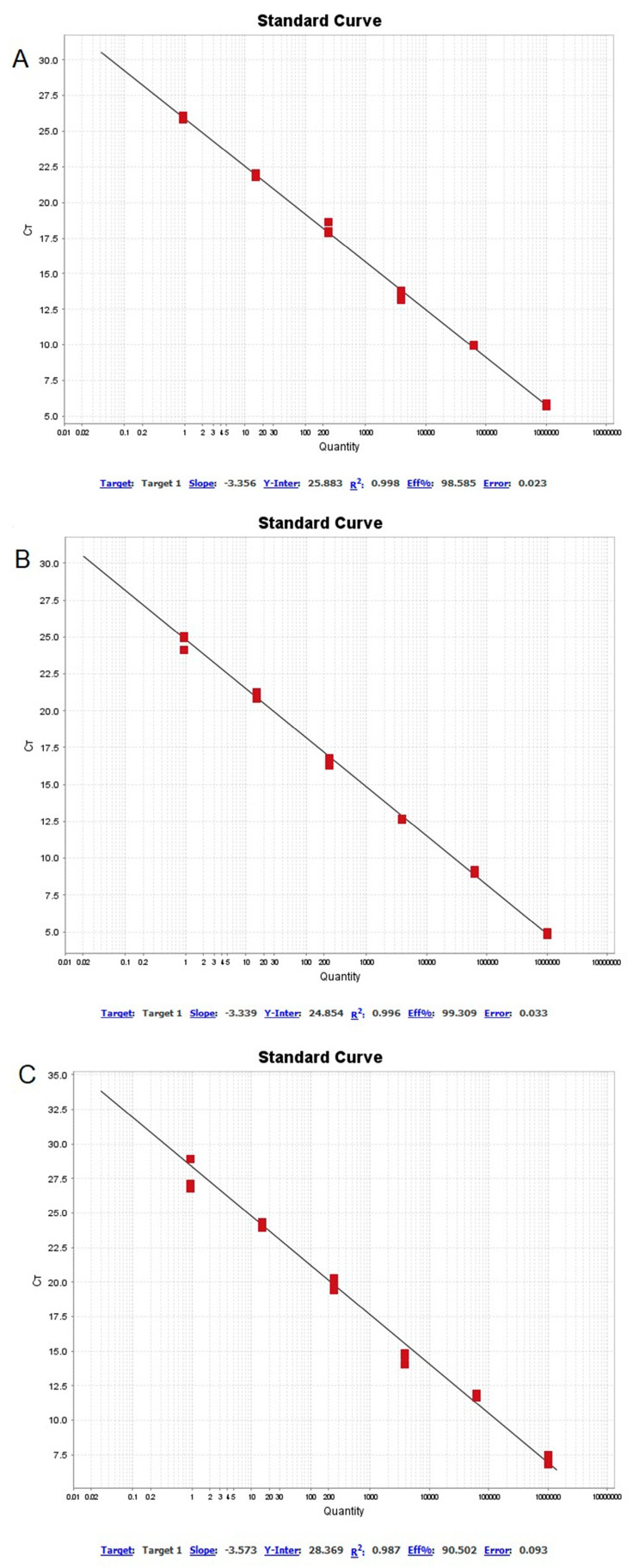
Standard curves were generated for three independent real-time PCR reactions: (**A**) species identification using *Francisella tularensis* subsp. *holarctica* samples, (**B**) species identification using *F. tularensis* subsp. *mediasiatica* samples, and (**C**) subspecies identification of *F. tularensis* subsp. *mediasiatica*. For each reaction, 16-fold serial dilutions of the samples were performed, starting with concentrations of 2.05 ng (1,000,000 genome equivalents, GE), 0.12 ng (62,500 GE), 8 pg (3906 GE), 0.5 pg (244 GE), 31.2 fg (15.2 GE), and 1.95 fg (0.95 GE).

The coefficient of determination (R^2^) for the real-time PCR detection of *Francisella* spp. was 0.998 for the *holarctica* DNA sample and 0.996 for the *mediasiatica* DNA sample. For the *mediasiatica* subspecies differentiation protocol, the R^2^ value was 0.987. The amplification efficiency of real-time PCR for *Francisella* spp. detection was 98.6% for *holarctica* and 99.3% for *mediasiatica* subspecies DNA, while the efficiency for *mediasiatica* subspecies differentiation was 90.5%. The slope of the regression curves for both protocols ranged from −3.339 to −3.573 (Figure 3).

### 3.3. Specificity Assessment

In the first stage, the specificity of real-time PCR with primers for the detection of *Francisella* spp. and subspecies differentiation of *F. tularensis* subsp. *mediasiatica* was tested on 86 DNA samples from non-*Francisella* spp. bacteria and three animal species. The collection of 86 bacterial species included isolates from environmental sources, as well as bacteria causing infections in humans and animals, including serologically cross-reactive bacteria such as *Brucella*. Real-time PCR showed no cross-reactivity of the selected primers, as indicated by the absence of an increase in the fluorescent signal during 45 cycles in wells containing DNA from heterologous bacterial and animal species. In the real-time PCR for subspecies identification of *mediasiatica*, no increase in the fluorescent signal was observed in the control DNA sample of *F. tularensis* subsp. *holarctica*.

In the second step, specificity was assessed on a collection of 28 and 39 DNAs isolated from *F. tularensis* subsp. *mediasiatica* and *F. tularensis* subsp. *holarctica*, respectively. In real-time PCR for the detection of *Francisella* spp., an increase in the fluorescent signal was recorded in all wells containing *Francisella* DNA samples, with samples containing *holarctica* subspecies DNA having a mean threshold cycle value of 14.522 (including the first 10 cycles excluding fluorescence) and *mediasiatica* subspecies having a mean threshold cycle value of 15.536 (Table 2 and Table S1, Figure S2A). The difference between the mean Ct values was greater than one cycle (*p*-value < 1.8 × 10^−10^). All samples were normalized to a single DNA concentration in the reaction (one ng). Examination of *F. tularensis* subsp. *mediasiatica* and *F. tularensis* subsp. *holarctica* DNA samples by real-time PCR for *mediasiatica* subspecies identification demonstrated primer specificity, as indicated by the absence of fluorescence increase in all samples containing *holarctica* DNA over 45 cycles. The average Ct value for *F. tularensis* subsp. *mediasiatica* DNA samples was 19.1 (Table 2 and Table S1, Figure S2B).

## 4. Discussion

Tularemia is an endemic infection in Kazakhstan, with over 10,000 human cases recorded during more than 90 years of monitoring. Most cases were reported between 1940 and 1980 during outbreaks that affected up to 1791 people, primarily linked to animal trade [42,43]. Annual monitoring of high-risk areas is a crucial aspect of tularemia control in Kazakhstan. Long-term surveillance of natural foci in endemic regions provides valuable data on the reactivation of tularemia before epidemic outbreaks, enabling timely epidemiological forecasts and the implementation of preventive measures [44]. Epidemiological monitoring, combined with vaccination campaigns in high-risk regions, has significantly reduced the number of cases. Between 1991 and 2015, 88 cases were registered, typically in sporadic outbreaks [45]. In the context of epidemiological monitoring in unfavorable territories, bacteriological, biological (infection of susceptible animals), and serological methods are employed to study suspensions of internal organs from dead or captured rodents and blood-sucking carriers. The bacteriological diagnostic method is labor-intensive and requires selective nutrient media. Consequently, the material collected from individual areas is often pooled [46]. This pooling can lead to diagnostic challenges due to sample dilution. Therefore, it is advisable to introduce screening methods based on easily reproducible and effective techniques. This strategy would enable the screening of non-pooled samples, with subsequent bacteriological analysis conducted only on positive samples. The isolation of pure bacterial cultures remains a necessary element in the study of virulence, resistance to antibacterial drugs, and whole-genome sequencing [47]. Although a non-cultivated and culture-free approach has been proposed for near-whole-genome sequencing of *F. tularensis* [48], serological methods for antigen detection exhibit low sensitivity [49] and can cross-react with *Brucella* and *Yersinia* [50], making them suboptimal for screening biological material from rodents and ticks. In this study, we developed a real-time PCR assay for the detection of *Francisella* spp. and subspecies identification of *F. tularensis* subsp. *mediasiatica*, for which the limit of detection (LOD95%) was 0.733 fg (0.358 genomic equivalents) and 8.156 fg (3.979 GE), respectively. The selected primers did not cross-react with DNA from 86 bacterial species and three animal species.

For real-time PCR detection of *Francisella* spp., primers were designed to target the ISFtu2 transposable element. ISFtu2 is 865 bp in length, belongs to the S5 family, is flanked by 19 bp terminal inverted repeats, and contains an open reading frame encoding a putative transposase [51]. ISFtu2 is present in varying copy numbers in three subspecies of *F. tularensis*, as well as in the genomes of *F. philomiragia*, *F. noatunensis*, and *F. novicida* [32,52]. It is often utilized in PCR testing of clinical animal materials and environmental samples to screen for *F. tularensis*, in addition to species- and subspecies-specific primers [53,54,55]. Several primer and fluorescent probe variants have been proposed for ISFtu2, with sensitivities ranging from four to less than one GE [56,57,58]. In our studies, the sensitivity of real-time PCR targeting ISFtu2 with *F. tularensis* subsp. *holarctica* DNA was four times higher than that with *mediasiatica* DNA. The detection limit (LOD95%) was five times lower with *holarctica* DNA. Furthermore, when testing specificity on 67 DNA samples, the cycle threshold (Ct) value for *mediasiatica* was consistently higher than that for *holarctica* at the same concentration in the reaction mixture. This discrepancy is primarily attributed to the number of ISFtu2 copies in the genomes: 42–44 copies per genome were identified in *F. tularensis* subsp. *holarctica* strains, while only 17 copies per genome were detected in *F. tularensis* subsp. *mediasiatica* strain FSC147 [59]. The efficiency of real-time PCR amplification for detecting *Francisella* spp. in DNA samples exceeded 98%, with an R^2^ value greater than 0.99 and 0.996. The slope of the regression curve ranged from −3.339 to −3.573, which is acceptable for qPCR [60].

A real-time PCR assay for subspecies identification was developed to target the junction between ISFtu1 and its 5′ flanking region identified in subspecies *mediasiatica*. The selection of this region for primer design, which integrates the mobile element ISFtu1, is optimal given that the average nucleotide identity among the three *F. tularensis* subspecies (*tularensis*, *holarctica*, and *mediasiatica*) is 99.7% [59]. The genetic differences are primarily due to homologous recombination events occurring between the two main mobile elements, ISFtu1 and ISFtu2 [61,62]. In a previously proposed real-time PCR assay for subspecies differentiation of *F. tularensis* subsp. *holarctica*, primers targeting the integration region of the ISFtu2 insertion were used [30], with a sensitivity of 100 CFU/L [32]. The primers proposed in this study detect two homologous regions in all published whole genome assemblies of *F. tularensis* subsp. *mediasiatica*, encompassing the three currently known sublineages and thus covering the established subspecific genetic diversity [17,41,63]. The detection limit (LOD95%) for real-time PCR species differentiation of *mediasiatica* was 3.4 fg or 1.67 GE, which allows its use for direct subspecies determination in clinical material after confirming the presence of *Francisella* spp. DNA.

The proposed qPCR does not fully meet the laboratory service requirements for studying biological material and environmental samples during epidemiological monitoring, as it enables only subspecies identification of *mediasiatica*. However, it can be utilized in conjunction with previously established qPCR protocols for subspecies identification of *holarctica* and *tularensis*.

## 5. Conclusions

Work has now begun on implementing the developed real-time PCR assay for testing samples collected during the annual tularemia monitoring in Kazakhstan. We believe that this practice will increase the number of positive samples by reducing the need for pooling or eliminating pooling altogether, allowing for the subsequent isolation of bacterial cultures.

## Figures and Tables

**Table 1:** Primer and TaqMan probe sets used for subspecies typing of *F. tularensis* subsp. *mediasiatica* and for the detection of *Francisella* spp.

Primer/Probe Name	Sequence 5′-3′	Length	Concentration in PCR (nM)	Target Subspecies/Genus
isftu-2_F_242	ACAAAACCCTGATTTACAAGAAGT	153 bp	800	*Francisella* spp.
isftu-2_R_396	CCTAAAGCATCAGTCATAGCAT		400	
isftu-2_Probes_331	FAM-CCAACTGATCTACCAATTGCTTG-BHQ1		400	
FtM_452000_F	TTGTTTCAACTGTTCTATACCTCTAA	215 bp	600	*F. tularensis* subsp. *mediasiatica*
FtM_452000_R	CTACCTCCGTACTCTCCAGATT		600	
FtM_452000_probe	FAM-TCCTATTGAAAAGGTTTGGGCT-BHQ1		400	

**Table 2:** Average Ct values for testing the specificity of protocols on *F. tularensis* DNA samples.

qPCR and Used Primers	The Average Ct Value for 39 *F. tularensis* subsp. *holarctica* DNA Samples Was Calculated Using 1 ng DNA in the Reaction. (Standard Error of the Mean (SEM))	The Average Ct Value for 28 *F. tularensis* subsp. *mediasiatica* DNA Samples Was Calculated Using 1 ng DNA in the Reaction (SEM).
qPCR for subspecies differential identification of *mediasiatica* using primers FtM_452000_F, FtM_452000_R and TaqMan probe FtM_452000_probe	Not detected	19.1 (0.09)
qPCR for the detection of *Francisella* spp. using primers isftu-2_F_242, isftu-2_R_396 and TaqMan probe isftu-2_Probes_331	14.522 (0.044)	15.536 (0.122)

## Data Availability

The original contributions presented in the study are included in the article/Supplementary Material, further inquiries can be directed to the corresponding authors.

## Supplementary Materials

The following supporting information can be downloaded at: https://www.mdpi.com/article/10.3390/microorganisms12112345/s1, Figure S1: Probit curve used to calculate the limit of detection (LOD95%); Figure S2: Evaluation of qPCR specificity for DNA of *F. tularensis* subsp. *mediasiatica* and *F. tularensis* subsp. *holarctica*; Table S1: Metadata of DNA samples used for specificity assessment; Table S2: Metadata for eight complete genomes of *F. tularensis* subsp. *mediasiatica*.
