# Supplementary material for: Development of a Real-Time PCR Assay for the Detection of Francisella spp. and the Identification of F. tularensis subsp. mediasiatica
Source: Microorganisms. 2024 Nov 16;12(11):2345. doi: 10.3390/microorganisms12112345 (PMC11596666; doi:10.3390/microorganisms12112345)
Supplement: Supplementary file 1 [file microorganisms-12-02345-s001.zip › Figure S1 probit analys.pdf]

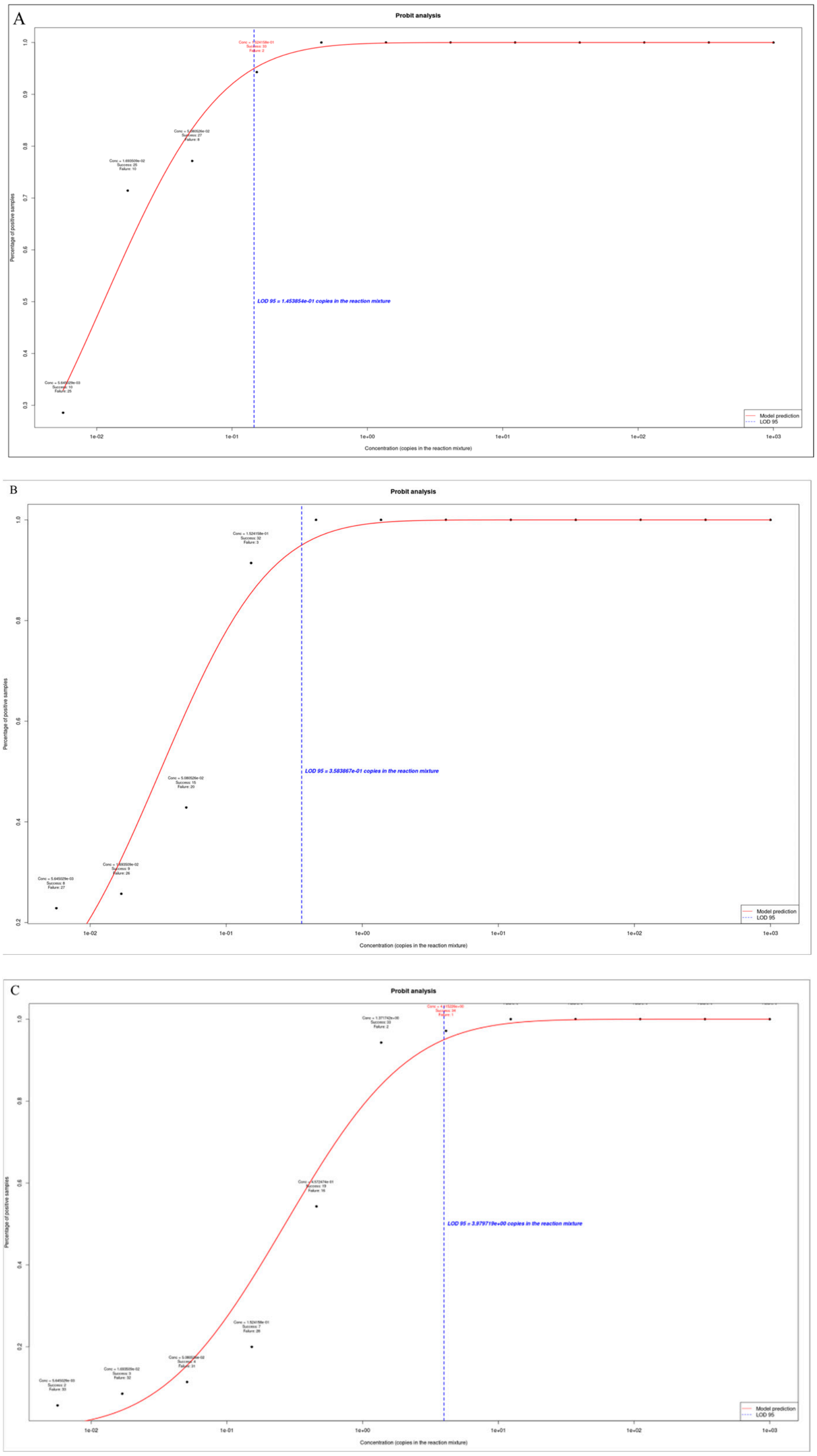

Figure S1. Probit curve used to calculate the limit of detection (LOD95%). A three-fold series of serial dilutions was performed DNA *Francisella tularensis* subsp. *holarctica* and *mediasiatica*, starting with an initial concentration of 0.002 ng (1000 GE) down to 0.01 fg (0.006 GE). Experiments were conducted in 7 repetitions over 5 days, for a total of 35 repetitions. The limit of detection at 95% was extrapolated from the sigmoid curve. A) LOD95% for real-time PCR detection of *Francisella* spp. using DNA *Francisella tularensis* subsp. *Holarctica B)* LOD95% for real-time PCR detection of *Francisella* spp. using DNA *Francisella tularensis* subsp. *mediasiatica; C)* LOD95% for real-time PCR of subspecies differentiation of *Francisella tularensis* subsp. *mediasiatica*
