# Supplementary material for: Development of a Real-Time PCR Assay for the Detection of Francisella spp. and the Identification of F. tularensis subsp. mediasiatica
Source: Microorganisms. 2024 Nov 16;12(11):2345. doi: 10.3390/microorganisms12112345 (PMC11596666; doi:10.3390/microorganisms12112345)
Supplement: Supplementary file 1 [file microorganisms-12-02345-s001.zip › Figure S2 Specificity on the collection of F. tularensis.pdf]

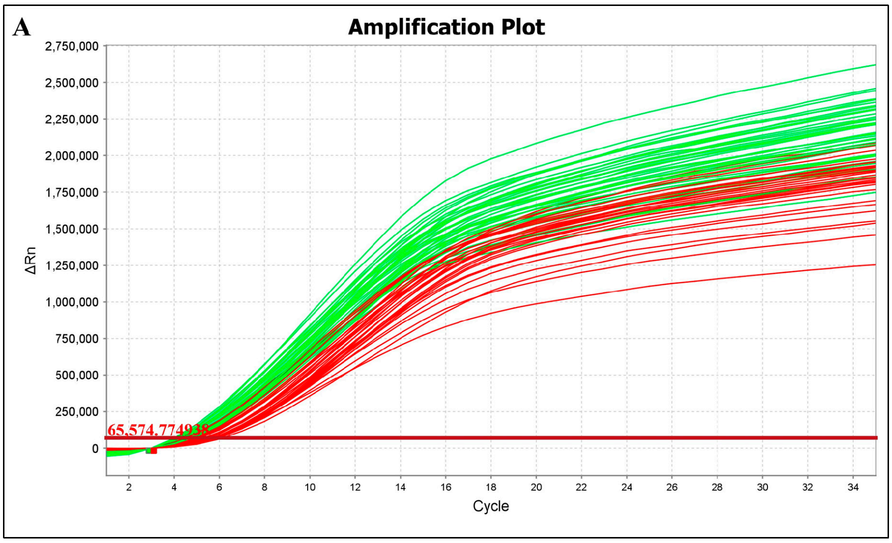

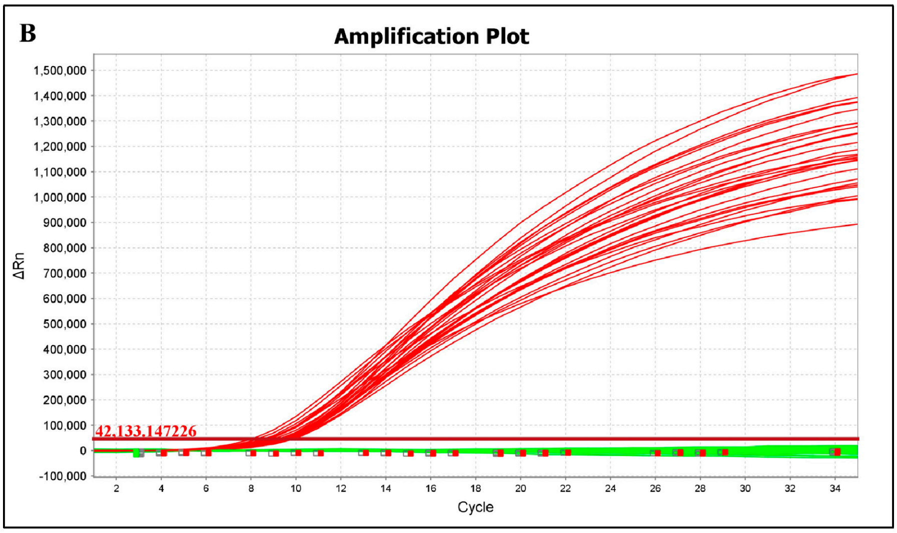

Figure S2. Evaluation of specificity on the collection of *F. tularensis* subsp. *mediasiatica* and *F. tularensis* subsp. *holarctica* DNA. A) Evaluation of the specificity of real-time PCR detection of *Francisella* spp. DNA of *F. tularensis* subsp. *holarctica* is highlighted in green, and DNA of *F. tularensis* subsp. *mediasiatica* is highlighted in red (Primers/TaqMan: isftu-2_F_242, isftu-2_R_396 and isftu-2_Probes_331). Each reaction contains 1 ng of DNA. B) Evaluation of the specificity of real-time PCR for subspecies differentiation of *F. tularensis* subsp. *mediasiatica* (Primers/TaqMan: FtM_452000_F, FtM_452000_R and FtM_452000_probe).
